# Mysterious Pelvic Hematoma in a Patient Who Speaks a Rare Ethiopian Dialect: A Case Report

**DOI:** 10.5811/cpcem.2022.10.57205

**Published:** 2022-12-29

**Authors:** Obianuju Eziolisa, Jennifer Chapman

**Affiliations:** Orange Park Medical Center, Department of Emergency Medicine, Orange Park, Florida

**Keywords:** spontaneous iliac vein rupture, iliac vein rupture, venous rupture, case report

## Abstract

**Introduction:**

In reporting this case of a patient with spontaneous iliac vein rupture, we highlight the importance of maintaining a high clinical suspicion of this vascular emergency in the at-risk patient.

**Case Report:**

A 50-year-old female with an uncommon language barrier presented with left lower abdominal pain after falling. Initial imaging showed pelvic hematoma of unclear etiology. Repeat computed tomography showed expanding hematoma, and after hemodynamic decompensation, exploratory laparotomy revealed a ruptured iliac vein.

**Conclusion:**

Although rare, spontaneous iliac vein rupture has a high mortality rate, even when identified early. This case serves as a reminder to consider this potentially fatal diagnosis in the at-risk group and highlights the need to remain vigilant in patients who present with unexplained shock. Additionally, this case is a reminder of our duty to provide emergency care that transcends language barriers.

## INTRODUCTION

Spontaneous iliac vein rupture (SIVR) is rare, with less than 50 confirmed cases reported worldwide. Iliac vein rupture is a vascular emergency usually due to trauma and/or iatrogenic cause. The exact etiology of SIVR is largely unknown, although several predisposing factors have been proposed. These include venous obstruction such as can be seen in May-Thurner syndrome (MTS) or Cockett syndrome, as well as deep vein thrombosis (DVT), thrombophlebitis, or other acquired or primary coagulopathy.^1^ Untreated iliac vein rupture can be lethal, due to catastrophic hemorrhage. However, the survival rate may be as high as 71–79% if immediately recognized.^2^ Many of these patients undergo exploratory laparotomy to manage unexplained hemorrhagic shock. In most cases of SIVR, the diagnosis is made during exploratory laparotomy. In this case report, we examine a case of SIVR presenting as hemorrhagic shock in a non-English-speaking, middle-aged Ethiopian female.

## CASE REPORT

An otherwise healthy 50-year-old female presented to the emergency department (ED) via ambulance after a ground-level fall. She was a non-English speaking Ethiopian who spoke only a native dialect that could not be translated with our services. This language barrier rendered her unable to communicate with the emergency medical services (EMS) workers, emergency physicians, or staff. She had fallen and called her son, who was not at home but who called EMS for her. Upon EMS arrival, the patient was sitting up on the bathroom floor. She was hypotensive with a systolic blood pressure around 90 millimeters of mercury, which improved after intravenous fluid bolus en route to the ED.

The patient’s son eventually arrived in the ED and provided additional history. Per her son, the patient worked in a factory and two days earlier had walked in the rain for about 15 minutes. She had been complaining of left buttock pain since her walk and was now complaining of left lower quadrant (LLQ) abdominal pain since her fall. She had no contributing past medical or surgical history. On exam, she had no signs of external trauma, including no abrasions, contusions, or other skin changes. Her abdomen was soft with tenderness to palpation in LLQ. Neurovascular exam was normal. The pelvis was stable. Extremities exam was normal with soft lower extremity compartments. The remainder of the physical exam was unrevealing. Initial hematology report revealed mild anemia with hemoglobin/hematocrit (H/H) 10.6/33.9 grams per deciliter (g/dL) (reference range: 11.2–15.7/34.1–44.9 g/dL), lactic acidosis of 4 millimoles per liter (mmol/L) (0.4–2.0 mmol/L), and mild acute renal injury. Chemistry panel was otherwise unremarkable.

**Image 1:**
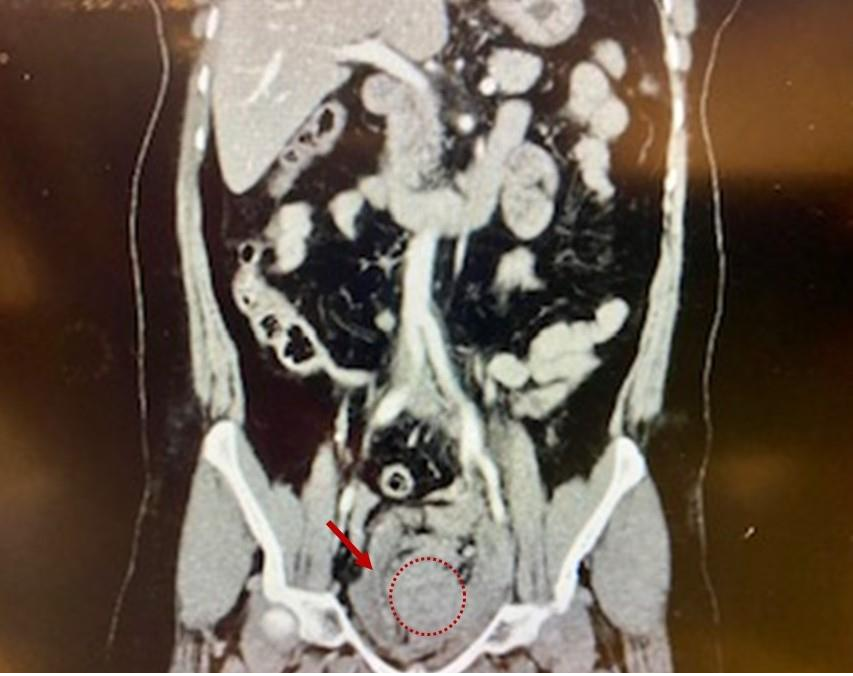
Initial computed tomography of the abdomen and pelvis, with pelvic hematoma compressing the bladder and ovaries. *Red arrow*: compressed and displaced bladder. *Dotted circle*: forming pelvic hematoma.

Initial computed tomography (CT) with contrast showed a pelvic hematoma with no associated fractures. Focused assessment with sonography in trauma exam was negative for free fluid; however, there was a large heterogenous-appearing mass in the suprapubic area with compression of the bladder similar to what was noted in the CT report (Image 1).

An urgent surgical consult was placed and cystogram was recommended to investigate for possible bladder injury. A foley catheter was inserted with no gross hematuria. During her ED course, she continued to have refractory hypotension despite continued fluid resuscitation. Approximately two hours after arrival, she experienced a vasovagal episode while sitting up and moving around on her stretcher. On re-examination, she was newly diaphoretic, pallid, and altered. Repeat H/H showed significant drop from 10.6/33.6 to 8.0/26.2. Emergent transfusion of two units packed red blood cells (pRBCs) was administered.

**Image 2:**
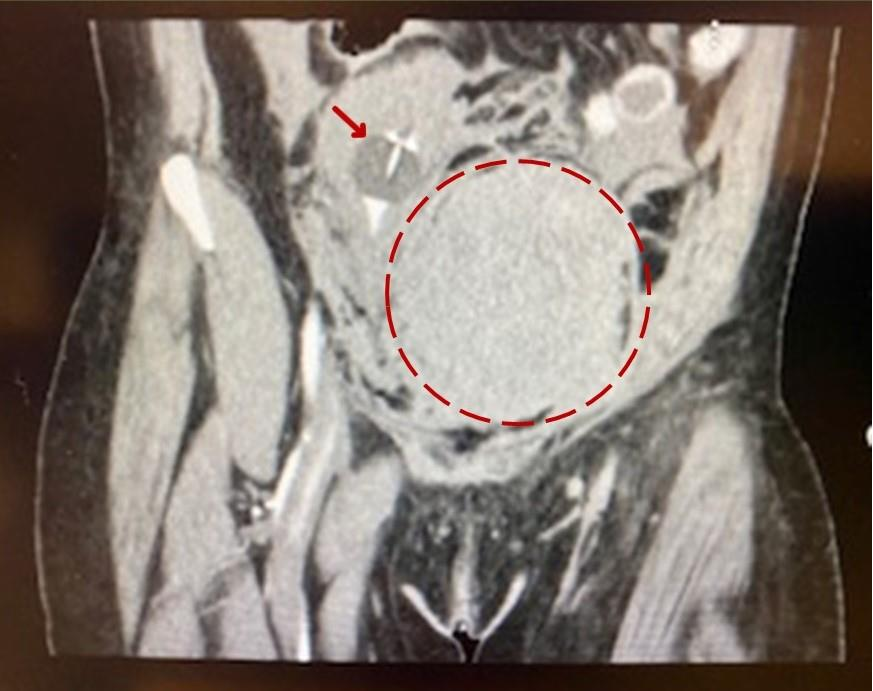
Repeat computed tomography of the abdomen and pelvis three hours later, with significantly expanded hematoma. Foley in compressed urinary bladder and bladder displaced. *Red arrow*: Foley in compressed urinary bladder and bladder displaced. *Dashed circle*: expanded pelvic hematoma.

The patient’s mental status improved, as did the physical finding of diaphoresis; however, she continued to complain of left lower extremity (LLE) pain. Repeat exam revealed new tense compartments of the entire left leg, with decreased sensation, motor function, and distal pulses in the left leg. On CT angiogram with runoff ordered to investigate arterial patency, the study revealed a significant increase in the size of the pelvic hematoma with mildly diminutive but patent caliber of the femoral arteries, tortuous external iliac, and common femoral veins, with findings suggestive of a likely venous or gynecological source (Image 2). The patient continued to be hemodynamically unstable despite additional transfusion of two units of pRBCs.

*CPC-EM Capsule*

What do we already know about this clinical entity?

*We know that spontaneous iliac vein rupture can be fatal if not identified and emergently managed. It is not a common diagnosis in the patient with abdominal pain and hypotension.*

What makes this presentation of disease reportable?

*We add one more ‘zebra’ to the differential. If untreated, this disease is fatal. Diagnosis is often delayed, contributing to the high morbidity associated with the disease.*

What is the major learning point?

*This case paints a picture of the typical demographic. It also serves as a reminder of the investigative nature of our specialty, one that has no barriers, be it cultural, language, or identity.*

How might this improve emergency medicine practice?

*The eye cannot see what the mind does not know.” We hope that dissemination of this case may lead to timely identification with the next presentation, and ultimately improvement of our patients.*

The decision was made to transport the patient to the operating room (OR) for exploratory laparotomy due to concern for continued intra-abdominal hemorrhage with no identified source. Intra-operatively, she underwent multiple rounds of massive transfusion protocol, and transfusion of frozen plasma for coagulopathy. In the OR, large retroperitoneal, preperitoneal, and pelvic hematomas were confirmed. Approximately one liter of clotted blood was removed from the pelvis. Repeated attempts at hemorrhage control were unsuccessful. Further exploration identified the source of bleeding: The iliac vein was visualized and found to have a large defect in the posterior medial aspect. The iliac vein was ligated successfully with resolution of bleeding. After hemostasis was achieved, tenseness of the LLE compartments resolved, thereby negating need for fasciotomy. Shock due to intra-abdominal hemorrhage resolved on postoperative day three, and the patient was discharged to home on postoperative day nine, on prophylactic aspirin therapy.

## DISCUSSION

Spontaneous iliac vein rupture, a rare vascular emergency, was first reported in 1961.^3^ Tannous et al^1^ published 33 cases and a literature review in 2006, and in 2010 Jiang et al^2^ published a case series that involved nine patients with suspected SIVR, although this was only confirmed by visualization or imaging in five of the nine patients.

Largely due to the rarity of SIVR, cases are often complicated by delay in diagnosis and associated with a high mortality rate of 26–29%.^1^ There is a higher prevalence in women, and the site of vessel compromise was left-sided in 94% of the cases reported by Hosn et al.^4^ The etiology of SIVR is likely multifactorial. Commonly postulated theories include mechanical factors, such as May-Thurner syndrome, stretched pelvic ligaments due to a multiparous state, inflammatory factors such as thrombophlebitis and DVT, as well as hormonal factors.^5^ Approximately 76–79% of cases were found to be associated with DVT and mechanical obstruction syndromes such as May-Thurner Syndrome.^6^ Other contributing factors include venous wall inflammation (due to multiple processes) and increases in intra-abdominal pressure (such as during the Valsalva maneuver). Once ruptured, the resultant hematoma may become large enough to tamponade the vessel, which may temporize blood loss.

We theorize that our patient presented with initial pelvic hematoma that created tamponade of the iliac vessel allowing for a brief period of stability. When she sat up in bed, the pelvic hematoma was disrupted and the vessel rebled causing acute decompensation.^7^ As the pelvic hematoma expanded rapidly, she developed acute venous congestion of the LLE leading to leg ischemia and phlegmasia.^8^ Approximately 30% of SIVR cases occur in patients with no known risk factors. Our patient had no known risk factors for SIVR, except post-menopausal age. While estrogen is pro-coagulopathic, there is a correlation with older females and SIVR.^1^ Both a lack of risk factors and the difficulty in communication made diagnosis difficult in this patient. Attentive care throughout the patient’s course including frequent reassessments and physical exams resulted in identification of rapid decompensation leading to early exploratory laparotomy and ultimate diagnosis.

The treatment options for SIVR hinge on appropriate and prompt diagnosis and vary depending on the patient’s hemodynamic status. Due to a paucity of cases, there is no uniform method of treatment, although Hosn et al^4^ published an algorithm of generally accepted options based on reported data of patient outcome. Essentially, if the pathology is quickly identified and the patient is hemodynamically stable, endovascular repair may be successful, while unstable patients require open repair. In this case, the source of bleeding was not clear on initial imaging and her rapid decompensation did not allow for further non-operative diagnostic work-up. This progression is typical of SIVR, with diagnosis often made during exploratory laparotomy. Definitive treatment options include repair of the defect or ligation of the vein. Venous bypass surgery (Palma-Dale procedure) may be performed to decrease likelihood of venous congestion.

## CONCLUSION

Spontaneous iliac vein rupture is a vascular emergency with high mortality and up to 50% morbidity.^9^ It can mimic other “can’t miss” pathologies such as ruptured abdominal aortic aneurysm, ectopic pregnancy, or other gynecological emergencies. Although rare, SIVR should be considered in the differential diagnosis of any older female complaining of left lower quadrant abdominal pain or left lower extremity pain and hypotension. The optimal diagnostic modality is a CT venogram, although a normal CT angiogram in the right clinical scenario can increase the index of suspicion. This case also highlights the importance of frequent examination in all patients, especially in the case of undifferentiated shock. Even more important is the need to maintain a high index of suspicion for dangerous pathology in individuals with barriers to care. This includes disability, age, and in this case, a language barrier. Sickness spares no one, and our clinical acumen must have no disparity.
